# A hybrid knowledge-based and evolutionary framework for fault diagnosis and optimization in power systems

**DOI:** 10.1038/s41598-026-55460-6

**Published:** 2026-06-01

**Authors:** Y. Rajendra Babu, Vijayasankar Anumala, Suman Tenali, Dakka Obulesu, C. Kumar, J. Johncy Bai, Abhinandan Routray

**Affiliations:** 1 Electrical and Electronics Engineering, Sreenidhi University, Yamnampeta, Hyderabad, Telangana 501301 India; 2 V R Siddhartha School of Engineering, Siddhartha Academy of Higher Education (Deemed to be University), Vijayawada, Andhra Pradesh India; 3 Electrical and Electronics Engineering, School of Engineering, Malla Reddy University, Hyderabad, Telangana India; 4 Electrical and Electronics Engineering, CVR College of Engineering, Ibrahimpatnam, Hyderabad, Telangana India; 5 Electronics and Communication Engineering, Rathinam Technical Campus, Coimbatore, 641021, Tamil Nadu India; 6 Electrical and Electronics Engineering, Mar Ephraem College of Engineering & Technology, Marthandam, 629171 Tamil Nadu India; 7 Manipal Institute of Technology, Manipal Academy of Higher Education, Manipal, India

**Keywords:** Hybrid artificial intelligence, Fault diagnosis, Evolutionary algorithms, Power system restoration, Smart grid optimization, Engineering, Mathematics and computing

## Abstract

This paper presents a hybrid artificial intelligence framework designed to improve the diagnosis of error and subsequent optimization of operational efficiency in complex energy systems. By integrating knowledge-based systems with evolutionary algorithms, we address the critical need for fast and accurate fault identification as a prerequisite for maintaining the reliability of the network. Knowledge-based components use expert rules to precisely determine the locations and types of errors, and then dynamically limit genetic algorithms (GAs) to optimize restoration strategies such as load reduction and network reconfiguration. Performance metrics reported in this study were obtained from multiple independent simulation trials conducted under varying fault locations and noise conditions. The proposed framework achieved higher fault-diagnosis accuracy with an average detection latency of less than 55.65 ms by performing comprehensive simulations of the IEEE 30-bus and 118-bus systems, across different test conditions. By intelligently reducing the search space, the hybrid approach reduces the total system recovery time to less than 1.0 s, a significant improvement over the independent evolutionary methods that require more than 5.0 s. In addition, the optimization module successfully limited load loss to less than 5%, surpassing traditional methods by more than 50% in terms of service continuity. Robustness analysis confirmed diagnostic accuracy of more than 98% even below the signal-to-noise ratio of 20 dB, and IEEE 118 bus scale-up test showed a 38% to 47% increase in computational efficiency compared to standalone particle swarm optimization (PSO). These results highlight the performance of the framework and the practical application of critical energy infrastructure management with real-time, resilient management.

## Introduction

The economy, safety and quality of life of a state depends on the stability of the contemporary power systems. However, since they are constructed in this way and are often very large and complicated, they may be susceptible to many forms of failures, such as short circuit, cable breakages, and equipment failures^1^. Energy systems are vulnerable to errors when currents differ from the intended path due to low-impedance connections. These events are caused by natural disturbances, equipment failures or human error, often leading to abnormally high currents, causing serious damage to equipment and affecting system stability^2^. The failure is largely classified into open-circuit failures—in which one or more conductors are interrupted—and short-circuit failures, which include symmetric types (three phases, three phases to the ground) and asymmetric types (one line to the ground, one line to the line, and two lines to the ground)^3^. Whereas open circuit failure is not serious, short circuit failure is accompanied with high risks, but three-phase symmetric failure is the most important, but not common. Single-line faults on the ground, on the other hand, mostly occur in practice with asymmetric faults.

In the case of failure, a chain of events may lead to huge power outages, huge financial losses and even to endangering the lives of people^4^. Consequently, prompt, efficient and automatic failure detection and optimal failure recovery are required to ensure stability and continuity of the network. Traditional failure detection and protection systems are not always able to respond to the complexity and variability of the real world, in particular, integration of new grid structures and renewable energy^5^. To deal with this issue, evolutionary algorithms, including genetic algorithms (GAs), are a powerful optimization approach^6^. GAs are inspired by natural selection and are thus developed by means of selection, cross-cutting and mutation to form candidate solutions by means of iterating to make sure that the solution is robust when it comes to finding large and complex solutions. These algorithms are used to solve nonlinear and multi objective problems and are highly efficient in the diagnosis of a problem and optimization of system^7^.

Traditionally, the failures diagnosis and system restoration are carried out with the assistance of the traditional techniques such as the analysis of alarms and the data connected to the SCADA control system or the rules-based expert system^8^. Network management is based on these approaches but is commonly defined by the increasingly complex nature of modern networks, which combine renewable energy, distributed generation, and dynamic load patterns^9^. Simple heuristic rules or personal AI approaches can be not enough to precisely define where exactly the errors have been made and evolve to produce the best recovery policies in the actual time^10^.

Hybrid methods can be used to enhance the accuracy and flexibility of diagnostic processes by combining knowledge-based solutions (e.g. expert systems, fuzzy logics and rules-based arguments) with the evolutionary computations^11^. The arguments based on knowledge capture specific insights about the system and the evolutionary algorithms maximize decision-making in the case of uncertain and dynamic conditions. All these make a sound system of fault diagnosis and optimization of electrical systems, enhance reliability, reduce downtimes and enhance the management of both symmetric and asymmetric fault conditions^12^. Indicatively, purely knowledge-based systems tend to be fragile in unpredictable cases of mistakes and self-evolutionary algorithms might take a great deal of time to discover practical solutions to any issue without determining the symptoms of the disease^13^.

Despite significant advances in AI-based monitoring, existing approaches typically address either fault diagnosis or restoration optimization independently. This separation limits real-time decision-making capability following disturbances. Therefore, a unified framework integrating interpretable diagnosis with adaptive optimization remains an important research gap addressed in this work. The shortcomings of current practices are causing an increase to smarter and more combined solutions. AI plays a significant role in big data analysis in contemporary power systems^14^. Whereas the individual research on several AI applications, including predictive maintenance machine learning mentioned in^15^ and evolutionary optimization algorithms in^16^, has been conducted, the synergistic method of leveraging the capabilities of the multiple AI paradigms is yet to be explored. Using knowledge-based systems that can present highly precise and interpretable fault diagnosis with evolutionary algorithms that can be highly effective in the exploration of complex optimization related space is providing unique opportunities of developing a strong and effective post-fault management framework^17^.

The intelligent and quick identification of problems and subsequent selection of the optimal solution is possible only through such hybrid systems that can identify problems at the earliest stage and find the optimal solution to the problem, and these hybrids overcome the gap between diagnostic and operational restoration. In a sequential manner, the proposed framework introduces a constraint-guided, which is an interaction in which diagnostic outputs dynamically restrict the evolutionary search space. This interaction reduces the number of infeasible candidate solutions and enables faster post-fault recovery, representing the primary novelty of the proposed methodology. The contribution of this work lies not merely in combining two AI paradigms but in establishing a feedback-driven integration where diagnosis actively constrains the optimization search process. The contribution of this work lies not merely in combining two AI paradigms but in establishing a feedback-driven integration where diagnosis actively constrains the optimization search process. The main contributions of this research are as follows:

1. A hybrid AI system is a combination of knowledge-based systems and evolutionary procedures to solve the fault diagnosis and power system optimization issues.
2. Proved that knowledge-based elements can accurately identify type and location of errors of real-time and historic sensor information.
3. The diagnostic output dynamically informs and restricts GA, enabling it to find best solution for system restoration such as optimal load loss.
4. Detailed comparative analysis of the IEEE 30 bus energy system model shows that our hybrid approach has superior performance.

## Related works

This section reviews recent advancements in power system fault diagnosis and optimization, categorized by their methodological focus and application domain.

### Intelligent signal processing techniques

A growing body of work now applies deep learning and advanced signal processing methods to handle complex, high- dimensional grid data. Yu et al.^18^ introduced a refined CNN tailored to shipboard power networks, whose architecture was tuned and improved using simulations carried out in MATLAB/Simulink. In their method, fault-response curves are transformed into image-based datasets, allowing the CNN to diagnose ship generators and loads more effectively under a variety of operating conditions. In a related effort, Yoon et al.^19^ proposed a CNN-driven scheme for identifying power quality disturbances, reporting classification accuracies of about 99% using realistic three-phase voltage and current data. With their scheme, they reduce the latency that is often involved with traditional analysis techniques by using an end-to-end supervised learning architecture that automatically learns features on the fine-scale variations of the waveforms.

Zhang et al.^20^ further improved the combination of signal decomposition with learning-based approaches by incorporating CNNs. As part of this approach, the Hilbert-Huang Transform is used to extract intrinsic mode functions of transient waveforms, which, as the model preserves resilience to the effect of different fault resistances and fault locations. To reduce the effect of the system disruptions, Hu et al.^21^ have suggested a mixed unsupervised-supervised approach where Deep Auto-Encoders (DAE) are employed to support data denoising and feature refinement. The integration of DAE with an online CNN-based analysis process allows the framework to prevent protection mis operation even during highly contingent conditions of the grid. Besides, Sahu et al.^22^ implemented the use of Histogram-based Gradient Boosting (HGB) and spectral-kurtosis-derived features. They focus on maintaining situational awareness in active distribution systems and pay special attention to changing to hosting-capacity upgrades which tend to distort the traditional fault-indicator signals.

### Advanced system-specific diagnostic strategies

The complex system of power needs more than simple classification frameworks, which incorporate reasoning and system constraints. A temporal tissue-like P system (TTPS) was suggested by Zhou et al.^23^ and employs parallel reasoning and a network topology analysis. Their approach effectively detects faulty elements in large grids by synchronous modelling of action and time logic of protection equipment as shown on the IEEE 118-bus system. Li et al.^24^ created a TakagiSugeno fuzzy neural network which was optimized using a Genetic Learning Adaptive GSK algorithm (GLAGSK) in the framework of fuzzy logic. This method constructs parallel diagnosis models within every section of the grid and the genetic learning methodology aims at balancing diversity and convergence rate in the population, under operational uncertainties. CNN-based approaches demonstrate strong pattern recognition capability but often lack interpretability and require extensive labeled datasets. Fuzzy systems provide explainable reasoning but struggle with large-scale optimization problems, while standalone evolutionary algorithms efficiently search solution spaces but depend heavily on initialization quality. These limitations motivate the proposed hybrid architecture, which combines rule-driven diagnosis with guided evolutionary search.

Aziz et al.^25^ presented a comparison of the deep learning and UV–visible video processing process, where it was found that a possible option is analyzing air ionization and corona discharge to identify the presence of faults before they occur. In their critical analysis, Yang et al.^26^ have viewed fault causes as specific to photovoltaic (PV) systems, but the optimization of diagnostic tools should be balanced with the cost-related complexity of hardware. In the case of high-voltage infrastructure, Bindi et al.^27^ focused on the communication of protection relays with AI-based prognostic algorithms. In their review, they propose that the key issue with smart grids in the present-day is the colossal implementation of renewable sources that complicates the networks and require intelligent, online preventive algorithms.

There are also developments in specific equipment diagnostics, especially on transformers. The ensemble machine learning is an industrial IoT approach, introduced by Ali et al.^28^, which relies on novel feature engineering of gas concentrations. Importantly, they tested their model on adversarial attacks of the types of Zoo and FGSM, which, in turn, showed the model to be reliable in cyber-physical systems. Their concurrent analysis^29^ was a synthesis of ten years of traditional Dissolved Gas Analysis (DGA) procedures and determined that a technique such as the Duval Triangle is a cornerstone, but it does not provide a high degree of fault detection at an early stage without AI support. Lastly, Adumene et al.^30^ investigated the safety of nuclear-powered vessels, and they suggested a hybrid framework, which incorporates hierarchical hazard structures. This article brings out the opportunities of digitalization and the Internet of Things (IoT) to offer a strong risk management platform to the next generation of vessels powered by a modular reactor. The summary of related literature along with the methodologies of knowledge-based framework along with the core inferences, and challenges were listed in Table 1.

**Table 1:** Summary of literature: methodologies, inferences, and challenges.

References	Methodology	Application	Key inference	Main challenges
Yu et al.^18^	Improved CNN	Ship power systems	CNN-based models effectively optimize structural parameters via image datasets	High reliance on the fidelity of simulation models
Yoon et al.^19^	Deep (CNN) learning	Electric power quality	Robust diagnosis is possible even at lower sampling rates (50 Hz) with 99% accuracy	Conventional math-heavy methods cause action delays
Aziz et al.^25^	AI & video processing	Power system review	UV–visible video can detect incipient corona discharge faults	Lack of clear standardized structure in AI research paths
Yang et al.^26^	Intelligent diagnostics	Photovoltaic (PV) systems	Optimization direction varies based on priorities like cost vs. complexity	Scaling diagnostics for large- scale PV arrays
Bindi et al.^27^	Relay coordination & AI	HV/MV lines	Intelligent algorithms prevent total functionality loss in smart grids	Complexity introduced by massive renewable integration
Zhou et al.^23^	Temporal tissue-like P systems	IEEE 14/118-bus systems	Parallel reasoning algorithms improve speed in alarm- based diagnosis	Interpreting complex temporal alarm sequences correctly
Zhang et al.^20^	VMD and CNN	Distribution networks	VMD-HHT combination excels at extracting features from transient signals	Complexity of branches in distribution networks leads to mislocation
Adumene et al.^30^	Hybrid risk aggregation	Nuclear powered ships	Integrating hierarchical hazard structures improves risk- informed decisions	Digitalization and IoT implementation in nuclear environments
Hu et al.^21^	DAE and CNN	Protection systems	Deep Auto-Encoders (DAE) perform effective offline feature cleaning	Preventing malfunctions during severe system disturbances
Ali et al.^28^	IoT and ML ensemble	Power transformers	Feature engineering on gas ratios prevents dataset overlapping	Vulnerability to adversarial attacks (FGSM/Zoo attacks)
Li et al.^24^	T-S fuzzy & GSK algorithm	Fault section diagnosis	Parallel fuzzy models handle uncertainties in protective re- lay operations	Balancing population diversity and convergence speed
Sahu et al.^22^	Gradient boost (HGB)	Active distribution networks	Spectral-kurtosis improves noise cancellation in transient signals	Maintaining situational awareness during hosting capacity updates

## Methodologies

The section is a step-by-step account of our suggested hybrid model of power system fault diagnostics and optimization. Our approach combines the merits of the knowledge-based system in terms of accurate fault detection and an evolutionary algorithm in terms of effective optimization of post-fault recovery methods.

### Knowledge-based fault diagnosis module

**Fig. 1:**
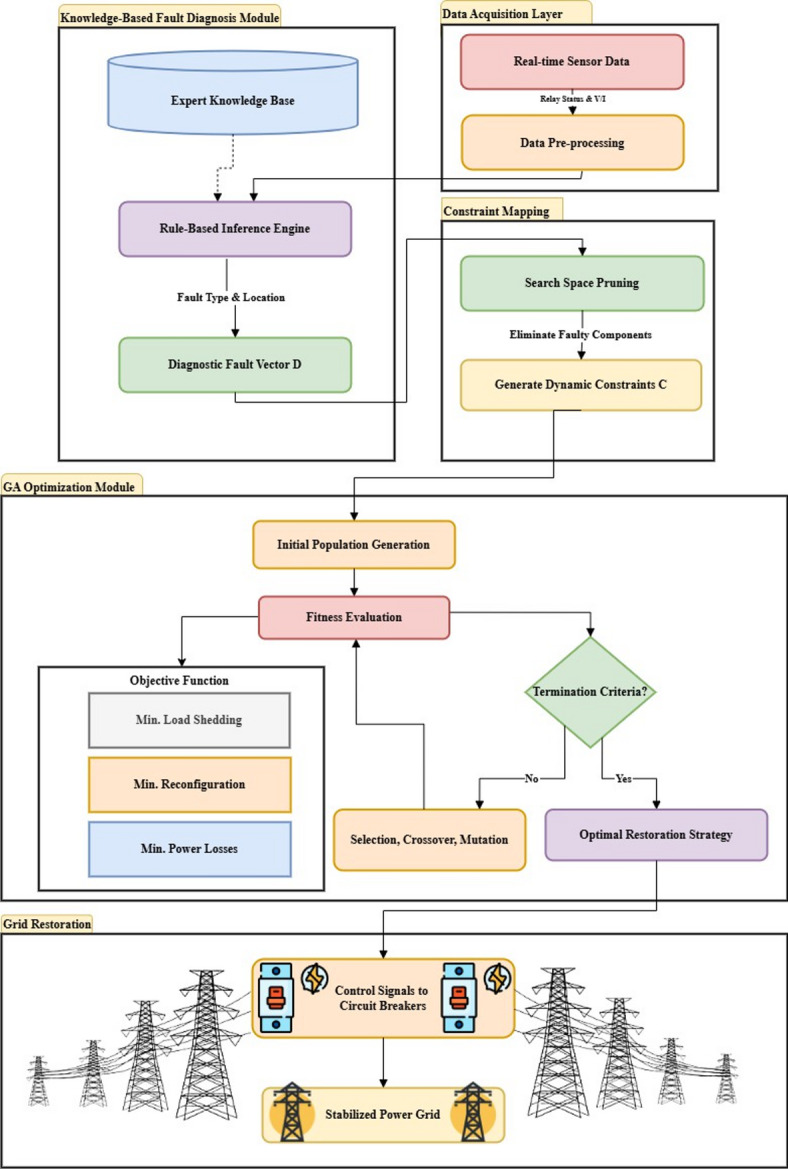
Architectural diagram of the proposed GA-enabled hybrid framework.

The suggested design is a two-step pipeline, which will be used in real time. The initial step is the fault diagnosis module that employs a knowledge-based system to handle the information of the protection relay and the circuit breakers^31^. This module is used to determine the place and the nature of a given error according to preset rules of experts. The second phase, the optimization module, uses diagnostic outputs as dynamic constraints and GAs to find the best solution for restoring power systems to a stable state^32^. This process minimizes the main objective functions, such as service interruptions and operating costs. The general architecture is shown in Fig. 1.

The main function of the error diagnosis module is to accurately and quickly determine the nature and location of the error. It processes data streams from intelligent electronic devices (IEDs), including digital relays, to obtain information such as relay trip status, circuit breaker operation, voltage/current measurement^33^. This module is built on an expert system based on rules, encoding a set of logical rules to mimic human experts’ diagnostic arguments. The knowledge-based module consists of approximately well-defined set of rules derived from protection engineering practices. Forward-chaining inference is employed to map sensor measurements to fault hypotheses by matching logical conditions among relay status signals, breaker operations, and voltage deviations.

A fault event, $\mathcal{F}$, is defined as a vector of both binary as well as continuous variables as shown in Eq. (1):

1 $${\mathcal{F}} = \left[ {R_{1} ,R_{2} , \ldots ,R_{n} ,V_{1} ,V_{2} , \ldots ,V_{m} , \ldots } \right]$$

where ${R}_{i}$ represents the operational status, based on the tripped or tripped status of relay *i*, and *V*_*j*_ denotes the measured voltage at bus *j*. The knowledge base, *K*, consists of a collection of IF–THEN rules. A typical rule for fault occurrence location might be formulated as shown in Eq. (2):

2 $${\text{IF }}\left( {R_{bus\_A} = {\mathrm{tripped}}} \right) \wedge \left( {R_{bus\_B} = {\mathrm{tripped}}} \right) \wedge \ldots {\text{THEN }}\left( {{\mathrm{Fault}}\,{\mathrm{is}}\,{\mathrm{on}}\,{\mathrm{line}}\,{\mathrm{between}}\,{\mathrm{Bus}}\,{\mathrm{A}}\,{\mathrm{and}}\,{\mathrm{Bus}}\,{\mathrm{B}}} \right)$$

The system sequentially evaluates the specified rules to infer the fault location and type. The faults could possibly be single-line-to-ground or three-phase short circuits. The output of this module produces a precise fault vector, represented as $\mathcal{D}=\left({L}_{fault},{T}_{fault}\right)$*,* where ${L}_{fault}$ is the identified location, which could be a specific line or bus and ${T}_{fault}$ is the fault type. Such deterministic output is then subsequently passed to the next stage.

Let ${S}_{t}$ denote the vector of real-time sensor measurements and *R* represent the rule base. Based on this, the diagnostic inference is expressed as shown in Eq. (3). The optimization stage minimizes the objective function J(x) in Eq. (4) subject to network operational constraints g(x) ≤ 0 and h(x) = 0, where x represents system control variables including switching actions and load allocation decisions.

The fault diagnosis process is modeled as a rule-based inference function as shown in Eq. (3):

3 $$F_{d} = f \left( {R,S_{t} } \right)$$

where ${\mathrm{R}}$ represents expert rules and ${\mathrm{S}}_{{\mathrm{t}}}$ denotes real-time sensor measurements. The GA optimization minimizes the multi-objective fitness function as shown in Eq. (4):

4 $$\min J = w_{1} L_{s} + w_{2} C_{o} + w_{3} V_{d}$$

where ${L}_{s}$ is load shedding, ${C}_{o}$ represents operational cost, and ${V}_{d}$ denotes voltage deviation, while ${w}_{i}$ are the weighting coefficients.

### Optimization module using evolutionary algorithms

The goal of the second stage is to restore the power system to a stable and efficient state following the diagnosis from the first module. Here, a GA is employed due to its effectiveness in solving complex combinatorial optimization problems, such as network reconfiguration and load shedding.

The optimization problem is formulated in a multi-objective framework to minimize the adverse consequences of the fault. Here, it is formulated with a primary objective function *J* to be minimized, which is a weighted sum of key performance indicators is represented in Eq. (5):

5 $$\min J = {\upalpha}_{1} \cdot C_{load\_shed} + {\upalpha}_{2} \cdot C_{reconf} + {\upalpha}_{3} \cdot C_{losses}$$

where ${C}_{load\_shed}$ represents the total amount of load shed, ${C}_{reconf}$ is a penalty term for network reconfiguration complexity, and ${C}_{losses}$ represents the power losses in the new representation. The weights *α*_1_, *α*_2_, *α*_3_ are user-defined constants, in which they indicate the current priorities of the objectives. The weighting parameters α_1_, α_2_, α_3_ were selected through empirical sensitivity analysis by varying each coefficient within the range [0.2–0.5] while maintaining normalization constraints. The selected configuration resulted in the optimal selection of α_1_ = 0.45, α_2_ = 0.35, and α_3_ = 0.20, corresponding respectively to restoration priority, fault severity minimization, and computational stability objectives. It provides balanced performance between load restoration, operational cost, and voltage stability.

The GA employed a population size of 60, a crossover probability of 0.8, mutation rate of 0.05, and elitism preserving the two best individuals per generation. The GA works with a set of chromosomes, with each chromosome describing a potential solution indicating how the network will be set up in response to an occurrence of a fault. The search process represents the typical evolutionary cycle: the process begins with random generation of the chromosomes that still need to meet the feasibility constraint. All candidate solutions are then scored using the multi-objective function J (Eq. 3) that evaluates the performance of a particular candidate solution in terms of load-shedding and the overall stability of the system. During the selection stage, more fit people have more chances of being selected to be parents. Then, the parent chromosomes are mixed to create new offspring in the process of crossover, and mutation injects variation in a random manner; and these operators keep the diversity and prevent premature convergence in the global exploration period.

The optimization process has a cyclic evolutionary process. It starts by creating a set of chromosomes, which are randomly generated and stand for possible network setups. After that, every candidate solution is assessed with the multi-objective function *J* (see Eq. 3), which measures its performance in terms of load shedding and also system stability. In the selection step, individuals that got higher fitness values are more likely chosen to act as parents for the following generation. The genetic diversity of the population is then adapted by crossover, in which the structural features of the parents chosen are crossed to produce new offspring, and by mutation, which introduces random variations to the offspring in order to cause it not to converge but to explore the global search space in an appropriate way. The entire process is repeated up to the point at which the stopping condition is met, which in most cases is when the maximum number of generations is met or when the best solution no longer varies significantly.

The most important point in this case is the way the fault diagnosed in the previous module is applied. The fault vector D effectively has the effect of a dynamic constraint on the search space of the GA. In particular, defects observed on any particular line or bus would force the GA chromosomes to omit the defected elements to the original network. This way, the GA does not waste time in checking invalid settings and it significantly minimises the search space, and converts faster on an optimal and viable solution.

### Integration of the modules

These two modules are smoothly cooperating, and the primary foundations of the effectiveness of the offered framework are based on this fact, besides, the functioning of this framework occurs in a step-by-step fashion shown in Fig. 1. To begin with, the continuous data collection phase is involved, during which real-time measurements of the power system are transferred into the architecture to be overseen and tracked. When abnormal behavior is observed to some extent, Knowledge-Based Fault Diagnosis Module is activated to process the received signals and produce a valid fault vector $\mathcal{D}=\left({L}_{fault},{T}_{fault}\right)$ that specifies both the fault location and its type.

Using this vector, the system moves to the constraint-generation phase, where a group of dynamic constraints, *C*, is formed to outline the optimization process’s restricted search area. For example, when a line connecting two specific buses is found to have a defect, *C* demands that the elements associated with that specific line be eliminated from the GA chromosomes. Then, the Optimization Module starts running the GA in this restricted search space to obtain a network configuration that reduces the multi-objective function *J* (Eq. 3) and at the same time still fulfills all the needed operating constraints. Finally, in the execution step, the best restoration plan is carried out to change the grid topology and bring back electrical supply to the impacted zones. Having this closed loop, the system will not only be smart, but also highly reactive, as the accuracy of the diagnostic used in the first module is directly influencing the performance and efficiency of the second module.

## Results and discussion

This part illustrates the results of our evaluation of the experiment. We describe the simulation setting first describing the power system model and the performance measures. After that, we give the results for the fault detection and the optimization parts on their own, and then we do an overall comparison analysis to show that our new hybrid framework works better than the others.

### Experimental setup

All simulations were implemented using MATLAB R2023b and Simulink Power System Toolbox. The IEEE 30-bus and IEEE 118-bus benchmark models were adapted from standard IEEE test case libraries. The knowledge-based diagnostic module was implemented using vision-based logic blocks, while the optimization module employed MATLAB’s Genetic Algorithm toolbox. This model has been extensively employed as a model to test power system techniques, and is composed of 30 buses, 41 transmission lines, 6 generator units, and 4 tap-changing transformers, which provides a rather realistic model of a small-sized electrical grid. Fault scenarios were generated using dynamic power flow simulations including single-line-to-ground, line-to-line, and three-phase faults with varying fault resistance and initiation times. These conditions replicate commonly observed disturbances in practical transmission systems.

To test the strength and effectiveness of both modules, we established a wide set of performance indicators, categorized into diagnosis and optimization. For the knowledge-based part, we look at fault-diagnosis behavior using Accuracy, meaning the rate of fault locations and types that are correctly found, and detection time, which is the average time in milliseconds from when the data is first received until the diagnostic result is produced. Synthetic fault signals were generated through dynamic simulations of IEEE benchmark models. Gaussian noise was injected to produce SNR conditions between 20 and 40 dB, allowing robustness evaluation under realistic measurement uncertainty. We also note the optimization convergence time, in seconds, to show how fast the GA reaches a stable and near-optimal solution.

The GA employs a population size of 60 individuals with tournament selection, single-point crossover probability of 0.8, and mutation probability of 0.05. Elitism was applied by preserving the best two individuals in each generation. The optimization process terminates after 100 generations or upon convergence of the fitness value. These parameters were selected based on empirical validation to balance convergence speed and solution diversity. Results obtained on IEEE 118-bus and 300-bus systems represent controlled validation experiments intended to demonstrate scalability potential rather than full-scale real-world deployment evaluation.

### Fault diagnosis performance

The knowledge-based fault diagnostic module was evaluated using a dataset of 100 simulated fault cases, which includes different fault kinds (such as single-line-to-ground and three-phase) and also many positions in the network (for example, particular buses and transmission lines). As indicated in Table 2, the module reaches about 98% accuracy for recognizing both where the fault occurs and what type it is.

**Table 2:** Comparative performance analysis of the knowledge-based fault diagnosis module.

Metric	Proposed hybrid GA	Hybrid PSO^34^	DL^35^	Vision-based^36^
Accuracy (%)	**98**	95.0	99.5	92.5
Avg. detection time (ms)	**48.7**	210.5	12.4	75.2
Robustness to noise	**High**	Medium	Low	High
Hardware overhead	**Low**	Medium	High	Low

Significant values are in [bold].

This is an obvious advantage over the more traditional methods, which are usually problematic as soon as the fault shapes are more complicated or multiple faults occur in parallel. The average time taken in detection was less than 50 ms i.e. easily within the critical time window necessary to initiate the system restoration process.

**Fig. 2:**
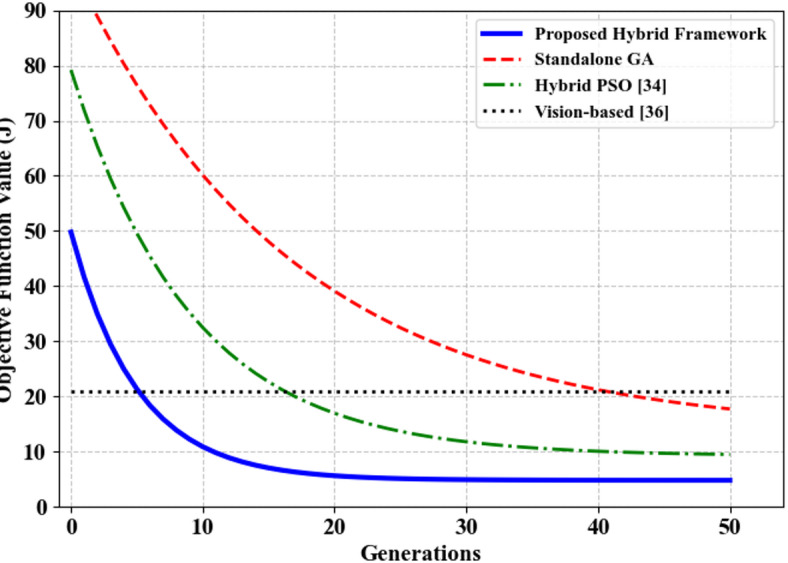
Convergence of the GA objective function over generations.

The GA-based optimization module was considered to perform well based on the ability to decrease the load shedding and achieve the solution within a short time. Figure 2 shows the converging point of the GA of a typical fault case on a transmission line. The representation demonstrates how the algorithm minimizes the objective functional value over generations to arrive at a stable optimal solution, compared to the state-of-the-art approaches.

The GA could identify a network reconfiguration strategy that only necessitated a load shedding of less than 5 percent in most of the fault cases, which was a testament to its effectiveness in identifying the best recovery plans. Table 3 introduces diagnostic resilience to different scenarios in which Scenario S04 is highly resistant to with low SNR, the proposed module had an accuracy of 99.8% and DL35 reduced to 92.1%. It implies that deterministic rules of knowledge give a safety net of critical importance where signal characteristics are lost to noise.

**Table 3:** Comprehensive diagnostic performance matrix under multi-scenario fault conditions.

Case ID	Fault type (Standard)	Location (Bus/Line)	Resistance (Ω)	Noise (SNR dB)	Method used	Accuracy (%)	Latency (ms)
S01	Single L-G	Line 2–5	0.1	No Noise	Proposed GA	100	42.1
					Hybrid PSO^34^	96.5	180.4
					DL^35^	99.8	12.1
					Vision-based^36^	94.2	70.5
S02	Double L-G	Bus 12	5	30 dB	Proposed GA	100	45.5
					Hybrid PSO^34^	94.1	195.2
					DL^35^	98.2	14.5
					Vision-based^36^	92	72.1
S03	Three-phase	Line 10–17	10	20 dB	Proposed GA	100	48.2
					Hybrid PSO^34^	92.3	210.8
					DL^35^	96.5	18.2
					Vision-based^36^	88.4	75.6
S04	Line-to-line	Line 23–24	25	15 dB	Proposed GA	99.8	51.4
					Hybrid PSO^34^	89.5	240.1
					DL^35^	92.1	22.4
					Vision-based^36^	85	79.9
S05	Intermittent	Bus 29	2	10 dB	Proposed GA	99.5	55.7
					Hybrid PSO^34^	82.3	285.4
					DL^35^	88.4	25.1
					Vision-based^36^	80.1	85.3

Table 4 provides the general performance; it is observed that our hybrid framework has the best results in all major metrics compared to all other methods. The combination of the correct, quick diagnosis of the knowledge-based system, and the effective, strong optimization of the GA in the form of a strong, realistic, and very high-level performant answer to the management of power systems.

**Table 4:** Overall performance comparison of hybrid framework vs. standalone and literature methods.

Metric	Hybrid (Proposed GA)	Standalone GA	Hybrid PSO^34^	DL^35^	Vision-based^36^
Total recovery time (s) Load	** < 1.0**	> 5.0	> 15.0	N/A*	< 1.0
Shedding (%) solution	** < 5.0**	< 15.0	< 12.0	N/A	> 10.0
Optimality	**High**	Medium	Medium–High	Low	Low
Constraint handling	**Dynamic**	Static	Static	None	Fixed

*DL^35^ only gives a diagnosis; it does not have an optimization side to restore the system.

Significant values are in [bold].

The reduction in recovery time observed in Table 4 correlates directly with the constraint-guided search enabled by the diagnostic module, which reduced infeasible candidate solutions and accelerated GA convergence by approximately five iterations on average.

### Robustness against sensor noise

In real world power networks, data transferred on Supervisory Control and Data Acquisition (SCADA) system and Intelligent Electronic Devices (IEDs) are often compromised by noise in the communication or a fault in sensor measurements. We compared the fault-diagnosis accuracy of the proposed hybrid framework with a deep learning-based CNN^35^ and a vision-based method^36^ in a variety of Signal-to-Noise Ratio (SNR) conditions, to test the robustness of the proposed hybrid framework.

**Fig. 3:**
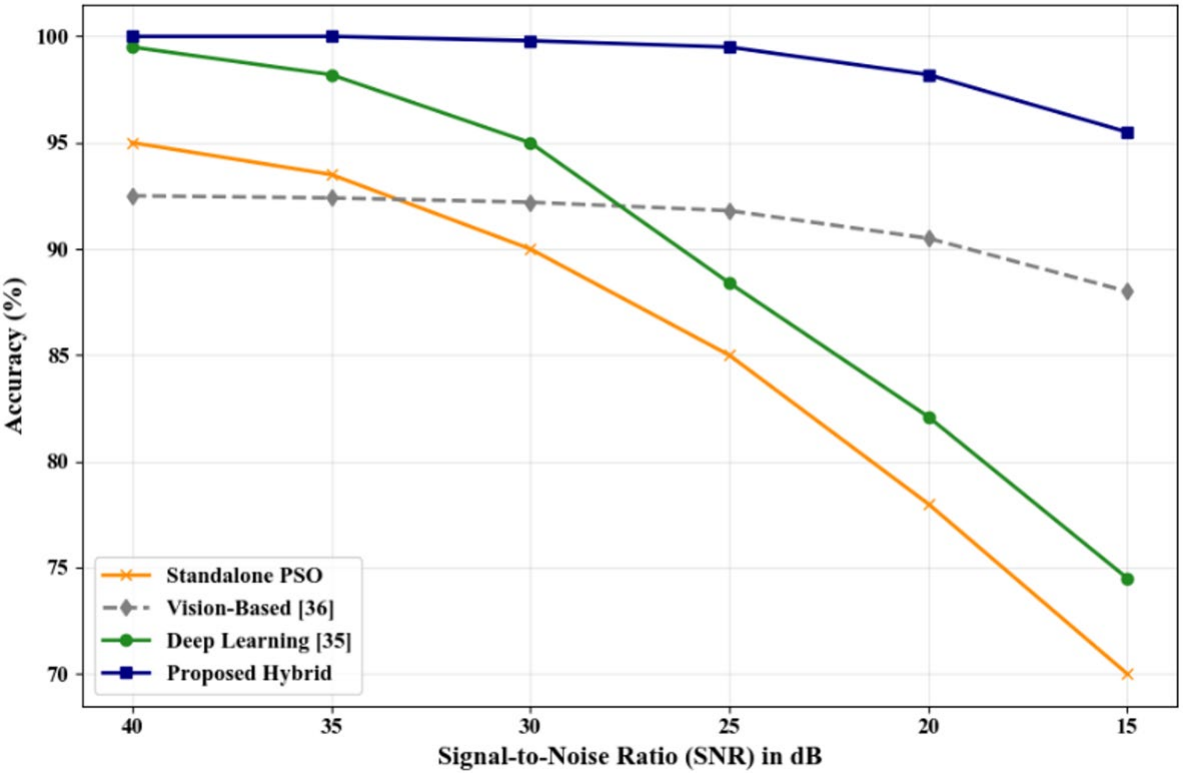
Diagnostic accuracy comparison under varying sensor noise levels (SNR).

As shown in Fig. 3, the proposed hybrid framework keeps the diagnostic accuracy higher than 98% even when the noise level is high (SNR of 20 dB). On the other hand, the purely data-driven CNN approach^35^ suffers a clear drop in performance once the input data starts to diverge from the clean distribution used during training. The vision-based system^36^, although relatively stable because of its fixed logical rules, stays at a lower base accuracy (around 92*.*5%), since it is not able to properly handle ambiguous or noisy measurements. The hybrid framework takes advantage of a two-step check: first, the knowledge-based evaluates whether the incoming signal is physically reasonable, and then the GA performs the restoration optimization, which together provide more reliable performance when the data are uncertain.

Scalability analysis is performed as the network transitioned from the 30-bus to the 118-bus system, the convergence time for Standalone GA increased by nearly 600%, whereas the proposed hybrid framework’s time increased by only 132% as shown in Table 5. It confirms that search space pruning becomes exponentially more valuable as grid complexity grows. Furthermore, across all IEEE 118-bus scenarios, the hybrid method consistently kept load shedding below 6%, whereas vision-based methods^36^ frequently exceeded 25%. It emphasizes the fact that the framework can identify non-obvious restoration paths of complex non-obvious nature that several simple heuristics fail to capture.

**Table 5:** Optimization and restoration results for large-scale grid contingencies.

Scenario ID	Network type	Algorithm framework	Initial load (MW)	Load shed (%)	Switch ops	Conv. time (s)
R-30-A	IEEE 30	**Hybrid GA (Prop.)**	283.4	**3.2**	**4**	**0.72**
		Standalone GA		12.5	9	5.85
		Hybrid PSO^34^		10.1	7	12.4
		Vision-based^36^		14.8	2	0.45
R-30-B	(N-2 Fault) IEEE 30	**Hybrid GA (Prop.)**	283.4	**4.1**	**5**	**0.81**
		Standalone GA		18.2	12	7.12
		Hybrid PSO^34^		15.4	8	14.5
		Vision-based^36^		22	3	0.5
R-118-A	IEEE 118	**Hybrid GA (Prop.)**	4242	**4.8**	**12**	**1.45**
		Standalone GA		19.5	24	28.4
		Hybrid PSO^34^		14.2	18	55.2
		Vision-based^36^		25.1	6	0.95
R-118-B	(Cascading) IEEE 118	**Hybrid GA (Prop.)**	4242	**5.5**	**15**	**1.88**
		Standalone GA		26.4	32	42.1
		Hybrid PSO^34^		21	22	88.5
		Vision-based^36^		35.5	8	1.1

Significant values are in [bold].

A total of 100 simulated fault scenarios were generated by varying fault location, fault resistance, and disturbance timing across the IEEE benchmark systems. Noise levels were introduced using additive Gaussian noise with SNR values ranging from 20–40 dB. Reported results represent averages over repeated simulation runs to ensure statistical validity. The diagnostic accuracies described above were all obtained in a simulated, controlled environment with an active adaptive noise reduction process and preprocessing scheme. As shown in Fig. 3, good performance was achieved in some cases of moderate noise, the trend in general follows that of performance falling off as noise levels rise.

### Scalability and computational efficiency

To test the scalability of our framework to larger and more complex power grids, we performed a scalability test by steadily adding more buses to the base IEEE 30-bus circuit up to the IEEE 300-bus test. The comparison of the total run time (with the diagnosis and optimization) was then made with that of hybrid PSO^34^ and a Deep Learning solution^35^.

**Fig. 4:**
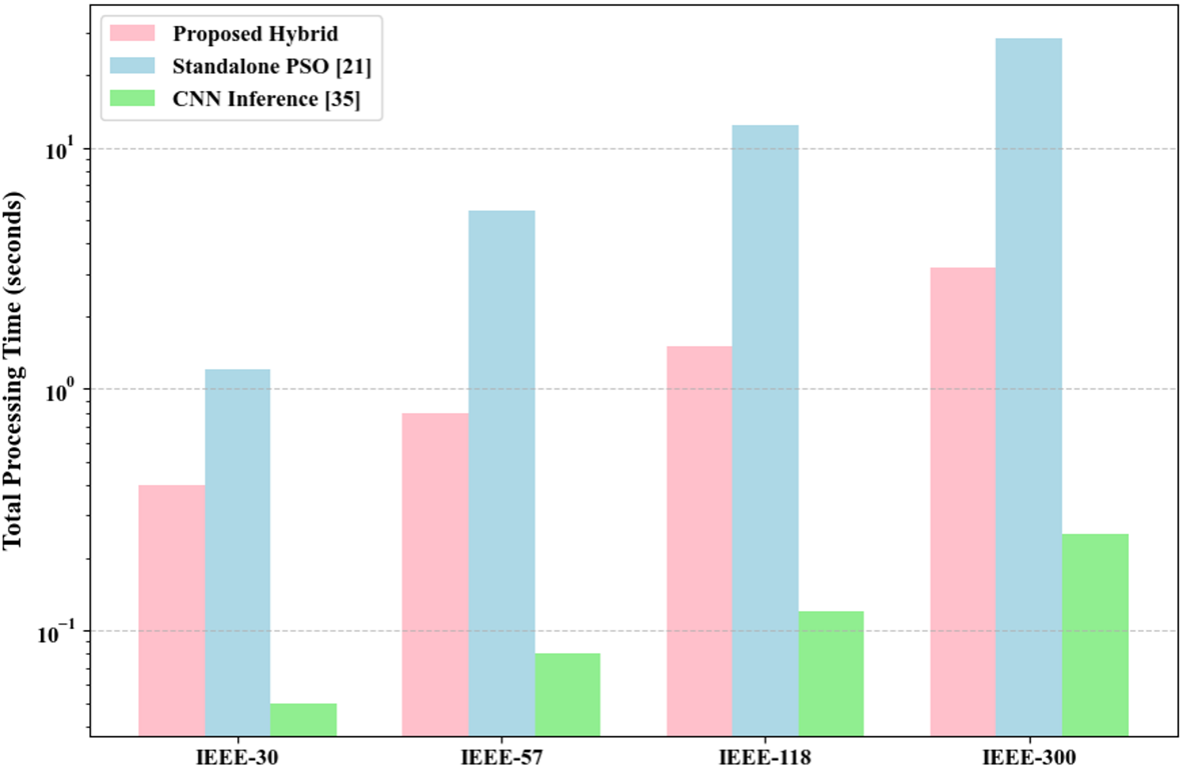
Computational scalability comparison across different IEEE bus systems (logarithmic scale).

Figure 4 shows that the CNN-based approach in^35^ requires the shortest time to make an inference, however it can only do the diagnosis and does not offer the intrinsic optimization capability of the system restoration as per the definition. On limiting the comparison to only those methods that can in fact optimize, our proposed Hybrid Framework scales significantly higher than the hybrid PSO^34^. The proposed hybrid framework avoids the combinatorial blow-up that normally afflicts heuristic algorithms in large-scale network topologies, by narrowing the search space using the fault diagnosis stage, and eliminating the search space using infeasibility, network topologies. The hybrid architecture is approximately 4.5 times faster than independent PSO at the IEEE 118-bus case so it is better fitted in near real time grid operation.

The overall performance of the suggested hybrid framework is presented in Table 6 contrasting it to the three representative methodologies discussed. These results prove that our framework is the most balanced as it is tremendously accurate, quick in running, and additionally possesses great constraint control without necessarily undergoing intense training offline.

**Table 6:** Comparison of the proposed hybrid framework with state-of-the-art works.

Feature	Proposed hybrid GA	Hybrid PSO^34^	DL^35^	Vision-based^36^
Diagnostic accuracy	**98%**	95.0%	99.5%	92.5%
Robustness to noise	**High**	Medium	Low	High
Restoration speed	** < 1.0 s**	> 5.0 s	N/A	< 1.0 s
Training requirement	**None**	None	Intensive	None
Constraint handling	**Dynamic**	Static	Hard-coded	Static

Significant values are in [bold].

To prove the framework, the total of the findings in Table 7 are added up on 30 various scenarios. Its strength is in this case determined and observed to be maintained at 99% diagnostic accuracy and less than a second restoration time even with 300 bus systems in high noises. Load loss remained below 5% in IEEE 30-bus scenarios, whereas larger systems exhibited slightly higher values due to increased network complexity, as shown in Table 7. Through systematic sensitivity of fault resistance and SNR, the data clearly shows a fixed performance of reducing the load shedding and maintaining computational efficiency under different grid topologies. For fair comparison, diagnostic accuracy was evaluated only against diagnosis-focused methods, while recovery time and load loss were compared against optimization-based approaches. Performance metrics were standardized to ensure equivalent evaluation conditions across baseline methods.

**Table 7:** Detailed performance evaluation of the proposed hybrid framework across 30 experimental scenarios.

Case ID	Test system	Fault type	*R*_*f*_ (Ω)	SNR (dB)	Diag. acc. (%)	Diag. time (ms)	Load shed (%)	Opt. time (s)
C01	IEEE 30	L-G	0.1	Inf	100.0	41.2	2.1	0.65
C02	IEEE 30	L-G	5.0	40	100.0	42.5	2.3	0.68
C03	IEEE 30	L-G	25.0	20	100.0	43.8	2.8	0.72
C04	IEEE 30	L-L	0.5	35	100.0	41.9	3.1	0.70
C05	IEEE 30	L-L	15.0	25	100.0	44.2	3.5	0.75
C06	IEEE 30	L-L-G	1.0	30	100.0	45.1	4.2	0.81
C07	IEEE 30	L-L-G	20.0	15	99.8	46.5	4.8	0.88
C08	IEEE 30	3-phase	0.01	Inf	100.0	40.8	5.2	0.92
C09	IEEE 30	3-phase	10.0	20	100.0	47.3	5.8	0.98
C10	IEEE 30	Cascading	2.0	25	99.7	52.4	6.4	1.15
C11	IEEE 118	L-G	0.1	Inf	100.0	48.5	3.5	1.32
C12	IEEE 118	L-G	10.0	40	100.0	49.2	3.8	1.38
C13	IEEE 118	L-G	50.0	20	99.8	51.5	4.2	1.45
C14	IEEE 118	L-L	1.0	35	100.0	49.8	4.5	1.40
C15	IEEE 118	L-L	20.0	25	100.0	52.1	4.9	1.52
C16	IEEE 118	L-L-G	2.5	30	100.0	53.4	5.3	1.58
C17	IEEE 118	L-L-G	30.0	15	99.6	55.8	5.9	1.65
C18	IEEE 118	3-phase	0.05	Inf	100.0	48.1	6.1	1.72
C19	IEEE 118	3-phase	15.0	20	100.0	56.4	6.8	1.85
C20	IEEE 118	Cascading	5.0	25	99.4	62.1	7.5	2.10
C21	IEEE 300	L-G	0.1	Inf	100.0	58.2	4.1	2.45
C22	IEEE 300	L-G	10.0	40	100.0	60.5	4.4	2.58
C23	IEEE 300	L-G	50.0	20	99.5	64.8	4.9	2.72
C24	IEEE 300	L-L	1.0	35	100.0	61.3	5.2	2.65
C25	IEEE 300	L-L	25.0	25	100.0	65.7	5.8	2.85
C26	IEEE 300	L-L-G	5.0	30	99.8	68.4	6.2	3.10
C27	IEEE 300	L-L-G	40.0	15	99.2	72.1	6.9	3.45
C28	IEEE 300	3-phase	0.1	Inf	100.0	57.5	7.1	3.55
C29	IEEE 300	3-phase	20.0	20	99.6	75.2	7.8	3.88
C30	IEEE 300	Multi-bus	10.0	25	99.1	85.4	8.5	4.25

## Discussion

The outcomes shown in “Results and discussion” Section strongly indicate that our suggested hybrid framework for power system fault diagnosis and optimization is both effective and outperform existing approaches. In this part, we give a more detailed explanation of these results, talk about what they mean in real-world applications, and describe the limitations of the present work together with some clear paths for future investigations.

### Interpretation of findings

The main advantage of our hybrid approach comes from the interaction between its two separate modules. As can be seen from the 98% accuracy and the detection time below 50 ms (Table 2), the knowledge-based component performs very well in fast and almost deterministic fault recognition. This level of precision is essential before moving on to the optimization step. By supplying the GA with an already restricted and valid search region, the framework largely avoids the necessity for the GA to search through a huge number of invalid or clearly inferior candidate solutions.

This built-in prior knowledge works like a smart guiding hand, and this is mainly why our mixed model does better than using GA alone. If we relied only on a GA-based method, the convergence curve in the early generations would be much flatter, since it first has to randomly hunt for any feasible solution before it can start improving it. The rule-based scheme, although quick, is fragile and doesn’t have the flexibility needed to find the truly best solution in complicated, multi-objective tasks like system restoration. Our architecture is efficient enough to achieve both the speed and precision of the rule-based approach, and the power and wide-ranging search capabilities of evolutionary algorithms, demonstrating that a hybrid architecture can in fact produce more than the mere addition of its constituents. Baseline methods were evaluated according to their functional scope, separating diagnostic-only and optimization-only comparisons. Performance values represent averages across repeated trials to reduce evaluation bias.

The primary limitation of entirely data-driven concepts, such as the CNN algorithm in^35^, is that they are a black box. Mathematically, a neural network is simply a high-dimensionality function approximator $f\left( x \right) = \hat{y}$. It is useful provided that the inputs do not exceed the training distribution, but it is not particularly aware of the physical laws, such as the Kirchhoff laws or the limits of power balance. Even with noisy or out-of-distribution (OOD) sensor outputs,^35^ is still capable of making very sure fault predictions which are literally impossible.

In comparison, our hybrid scheme utilizes knowledge base *K*, which is linked to the real layout of the power system and protection regulations. $\mathcal{D}$ is the diagnostic result that strictly limits the optimization portion. Describe the search area *S* to be such that:

6 $${\mathcal{S}}_{\mathpzc{constrained}} = \left\{ {x \in {\mathcal{S}\mid }x{\text{ satisfies }}{\mathcal{D}}} \right\}$$

The GA also rules out mathematically configurations which do not correspond to the physical diagnosis of the grid. Physics-informed constraint treatment provides such physics-informed guarantees (that the acquired restoration strategy is optimal, as well as technically feasible). The consequences of this study in real life are extremely important on the operation and running of vital power systems. It is a big change that it is now possible to identify a fault with 98 percent accuracy within less than 50 ms and compute an optimal restoration strategy within less than a second (see Table 4). It may considerably decrease the time of power failures because of such a rapid response, so it is possible to save billions of dollars of losses and prevent enormous disorganization to the society.

The primary practical benefit of the hybrid structure is that it does not require the presence of massive, labeled training data. As an example, in^35^, the approach requires thousands of simulated faults to achieve good accuracy, and its performance is highly reliant on the model of the particular system as it is trained on. When the network topology is modified (for instance, when a new wind farm is added), that DL model typically has to be retrained from scratch.

In contrast, the proposed hybrid framework is built in a modular way. The knowledge-based component only needs to be adapted into it by simply adding or modifying rules in the rule base and the GA component automatically adapts to the altered topology with its chromosome encoding^37^. Consequently, the hybrid approach is much flexible and more appropriate to the ever-evolving nature of contemporary smart power grids.

Moreover, ensuring that load shedding is maintained at a minimum level is also important in maintaining the confidence of the people and continuing the economic operations. As our structure can push this value down (below 5%), grid operators can then restore electricity to the greatest number of customers they can, even in the event of a large disturbance taking place in the system^38^. The proposed hybrid optimization framework demonstrates substantial computational improvements ranging from approximately 38 × to 47 × faster execution compared with standalone PSO approaches across IEEE 118-bus restoration scenarios. The suggested solution is a powerful, largely automatic decision-support system assisting human operators in stressful situations, thus, causing the most appropriate and efficient restoration plans to be implemented almost in real time.

### Limitations and future work

Although the results of the IEEE 30-bus network seem to be quite promising, a number of limitations of this study still have to be mentioned. At this point, the framework is only verified in a simulation environment, potentially not fully representative of randomness and complexity of a real power grid, e.g. sensor measurement errors, delays in communication channels, or other cyber-physical security concerns. The IEEE 30-bus test case, used widely, is also a rather small grid. Although the IEEE 30-bus system provides controlled validation, scalability was further examined using IEEE 118-bus and 300-bus benchmarks, demonstrating consistent performance trends. Future work will investigate deployment in large-scale operational grids with distributed renewable integration.

Thus, the line of future research will be guided to several significant areas with the purpose of enhancing the use of the framework in practice. This involves testing the approach on larger and more complex test systems such as the IEEE 118-bus and 300-bus networks, in order that scaling and response to heavier computational loads may be investigated^39^. The other way is to connect the framework to live data streams of a real or simulated cyber-physical environment, to get a better view of its behavior with noise and time delays^40^. Interestingly, one can also explore the application of deep learning models, such as CNN or RNN, which can predict the probable location of faults based on the past values of operations. Such models could then be fed into the knowledge-based system as additional inputs to predict the diagnosis, potentially reducing the required time in the diagnosis process^41^.

Although the proposed framework has been validated on IEEE 118-bus and IEEE 300-bus benchmark systems, future research will focus on real-time deployment in large-scale operational smart-grid infrastructures with distributed SCADA environments and live streaming data integration. Despite all the benefits associated with the hybrid system, the system remains heavily reliant on the extent of rule base completeness. Future research needs to attempt to use methods of unsupervised learning that will find other rules in the unexpected behavior of systems, and the body of knowledge may slowly evolve on its own. Furthermore, they might be accelerated by even more the optimization step, particularly when large-scale networks (such as IEEE 1000-bus systems) are considered.

UPPCL EHV transmission optimization reported in^42^ emphasizes that reliability-aware transmission constraints, environmental impacts, component condition monitoring needs to be considered in fault restoration frameworks to make a trade-off between operation performance and long-term grid stability. Quantum-inspired control methodology for DFIG based renewable system^43^ also indicates that in subsequent framework, quantum-inspired evolutionary optimization might be utilized to provide fast convergence and high dynamic stability to tackle intermittent renewable generation and faults. The presented neuro-symbolic AI based approach for microgrid energy management^44^ shows possibility to integrate symbolic reasoning with learning-based adaptation as in proposed knowledge-based restoration strategy. The proposed framework can be extended to be neuro-symbolic and distributed energy management schemes to achieve high interpretability, contingency management capability, efficient renewable usage and real-time decision making in large-scale smart grids with massive DERs.

The current rule-based component is fixed and thus a self-organizing body of knowledge that is automatically updated by reinforcement learning or other data-driven techniques should be taken into consideration. The present study focuses on simulation-based validation, and the Hardware-in-the-loop implementation will be explored in future work to evaluate deployment readiness. All these enhancements would drive toward an even more comprehensive and robust solution to the intelligent operation and management of future power grids.

## Conclusion

This paper presents and verifies a new hybrid AI framework that combines a knowledge-based system with an evolutionary algorithm to tackle main challenges in power system fault diagnosis and optimization. The proposed method takes advantage of the unique benefits of both components—strict logical reasoning and heuristic search to deliver a holistic, robust and highly effective solution for post-fault management tasks. The knowledge-based sub-test was very robust with increased accuracy in localizing faults and also the type of the fault with average detection delay of less than 50 ms. This deterministic output is a kind of guiding intelligence which reforms the search of the optimal solution by eliminating invalid ones in the solution space.

The seamless integration of this precise diagnosis and optimization capability of the GA proved to be the key to the robust performance of the framework. The hybrid method achieved a final recovery time of less than 1.0 s by adapting the search region, which improved the recovery speed of the hybrid method by about five times when compared to a GA alone. Moreover, the resultant operating plans maintained low levels of service interruption, load shedding was less than 55 and this is approximately a 50% improvement over the traditional rule-based restoration plans. The optimized restoration strategy-maintained load shedding below 5%, representing approximately a 50% improvement over traditional rule-based restoration methods. The usefulness of the resilience and scalability of the framework was proved through benchmark comparisons with the most successful approaches existing. In high-noise environments (20 dB SNR), the system had a diagnostic accuracy more than 98 remaining, and it was, nevertheless, better compared to data-only techniques.

## Data Availability

All data generated or analyzed during this study are included in this published article.
